# Remarkable N_2_O emissions by draining fallow paddy soil and close link to the ammonium-oxidizing archaea communities

**DOI:** 10.1038/s41598-019-39465-y

**Published:** 2019-02-22

**Authors:** Ling Wang, Kun Li, Rong Sheng, Zhaohua Li, Wenxue Wei

**Affiliations:** 1 Faculty of Resources and Environmental Science, Hubei University, Wuhan, 430062 China; 2 Key Laboratory of Agro-ecological Processes in Subtropical Regions and Taoyuan Station of Agro-Ecology Research, Institute of Subtropical Agriculture, Chinese Academy of Sciences, Changsha, 410125 China

## Abstract

Fallow paddies experience natural flooding and draining water status due to rainfall and evaporation, which could induce considerable nitrous oxide (N_2_O) emissions and need to be studied specially. In this study, intact soil columns were collected from a fallow paddy field and the flooding-draining process was simulated in a microcosm experiment. The results showed that both N_2_O concentrations in the soil and N_2_O emission rates were negligible during flooding period, which were greatly elevated by draining the fallow paddy soil. The remarkable N_2_O concentrations in the soil and N_2_O emission/h during draining both had significant relationships with the *Arch-amoA* gene (*P* < 0.01) but not the *Bac-amoA*, *narG*, *nirK*, *nirS*, and *nosZ* genes, indicating that the ammonium-oxidizing archaea (AOA) might be the important players in soil N_2_O net production and emissions after draining. Moreover, we observed that N_2_O concentrations in the upper soil layers (0–10 cm) were not significantly different from that in the 10–20 cm layer under draining condition (*P* > 0.05). However, the number of AOA and the nitrification substrate (NH_4_^+^-N) in the 0–10 cm layer were significantly higher than in the 10–20 cm layer (*P* < 0.01), indicating N_2_O production in the 0–10 cm layer might be higher than the measured concentration and would contribute considerably to N_2_O emissions as shorter distance of gas diffusion to the soil surface.

## Introduction

Nitrous oxide (N_2_O), one of the important contributors to the radiative forcing by greenhouse gasses (GHGs), is expected to remain the largest emission throughout the 21st century and greatly contributes to stratospheric ozone destruction^1^. The latest reports by the world meteorological organization indicate that the global mean surface mole fractions for N_2_O were 328.9 ± 0.1 ppb in 2016, which is approximately 122% of the pre-industrial level^2^. Rice paddies are primary sources of atmospheric N_2_O, accounting for approximately 11% of global agricultural N_2_O emissions^3^. China has a large area of rice paddy fields, and the annual total planting area of rice was 30.9 million hectares in China, which was 19% of the global rice planting area in 2014^4^. Furthermore, the total N_2_O emission from paddy fields in China is estimated to be 180 Gg yr^−1^ ^5^.

In rice-based ecosystems, the midseason drainage and dry-wet alternation in the rice growing season usually cause obvious N_2_O emissions, whereas the continuously flooded fields emit less N_2_O into the atmosphere^5–7^. Therefore, the water content and related oxygen availability are considered key factors affecting soil N_2_O emissions. Several studies found that the optimum water condition for N_2_O production was 60–70% water-filled pore space (WFPS)^8,9^. Furthermore, N_2_O emissions exponentially increased with decreases in soil WFPS from 90% to 70%, whereas decreases were observed when the WFPS declined from 70% to 40%^10^. N_2_O production in the soil is mediated by microbial processes, mainly nitrification and denitrification^11^. Ammonia-oxidizing microorganisms (AOM), including ammonium-oxidizing archaea (encoded by Arch-*amoA* gene) and bacteria (encoded by Bac-*amoA* gene), play an important role in the nitrification of rice paddy soil and N_2_O production process^12^. Denitrification includes the reduction of nitrate (NO_3_^−^), nitrite (NO_2_^−^), nitric oxide (NO), and N_2_O which are transformed by the *narG* and *napA*, *nirS* and *nirK*, *qnorB* and *cnorB*, and *nosZ* genes of denitrifiers, respectively^13^. By changing the soil aeration (O_2_) and redox state, soil moisture has a great effect on enzyme activities of nitrification and denitrification process, thus regulating soil N_2_O dynamics^14,15^. Numerous studies have investigated the relationship between N_2_O emission and the activities of functional nitrifiers and denitrifiers in paddy soil under different water conditions. Paddy soil in draining was shown to harbor significantly higher copy numbers of denitrifiers than those found in flooded soil, and functional genes of denitrifiers were closely linked to large N_2_O fluxes^13,16,17^. In addition, other studies have shown that the abundance of nitrifiers (Arch-*amoA* and Bac-*amoA* gene) in draining paddy soil was considerably more than that in flooded soil, and *amoA* gene abundance was significantly correlated to N_2_O emission rates^12,18^. These varying results imply that the importance of nitrifiers, denitrifiers, or both to N_2_O emission in draining paddy soil might differ between cases. However, most studies on the microbial regulation of N_2_O emissions from paddy soil have been carried out during the cropping season. Consequently, less attention has been payed to paddy soil in the fallow season only^19^, which is also an important period of rice paddy cultivation.

Traditionally, the fallow period of the double-rice fields is in the winter from November to April of the next year, and these fields are often submerged after heavy or prolonged rains^20^. Furthermore, without human management, the soil water conditions of fallow paddies mainly depend on the rainfall and atmospheric evaporation, resulting in fluctuations in soil moisture of fallow paddies. Such prolonged periods and great variation in soil water condition would induce considerable N_2_O emissions from fallow paddy fields. Previous studies reported that more than 33% of annual N_2_O emission occurred during the winter fallow season in Yintan, Jiangxi Province^20^, and earlier field observations revealed that annual N_2_O emissions as high as 60% to 90% occurred during the winter fallow season in Taoyuan, Hunan Province^21^, indicating the importance of N_2_O emission during that period. Nonetheless, studies on the microbial process and regulatory mechanisms of N_2_O production and emission under different water status in fallow paddy soil are scarce.

Therefore, in our study, soil samples were collected from a fallow paddy field in Huanghua, Hunan Province, and molecular methods were used to investigate the changes of nitrifiers and denitrifiers to flooding-draining practice in fallow paddy soil and the associated N_2_O emissions patterns. To reflect the soil properties and structure *in situ*, intact soil columns were used as experimental materials. Previous studies showed that intact soil responded with higher sensitivity to water changes than sieved soil did, and it also discharged more N_2_O^22,23^. Our objectives were to investigate (1) the patterns of N_2_O concentration and emission in fallow paddy soil under flooding and draining conditions, (2) the changes of nitrifying and denitrifying microbial communities to flooding and draining practices, and (3) the links between the N_2_O dynamics and changes in functional communities and the effects of soil water status. We hypothesized that the flooding and draining process would induce different patterns of N_2_O net production and emission in fallow paddy soil and different responses by nitrifiers and denitrifiers.

## Results

### Dynamics of N_2_O concentrations in soil and N_2_O emissions from soil surface

**Figure 1:**
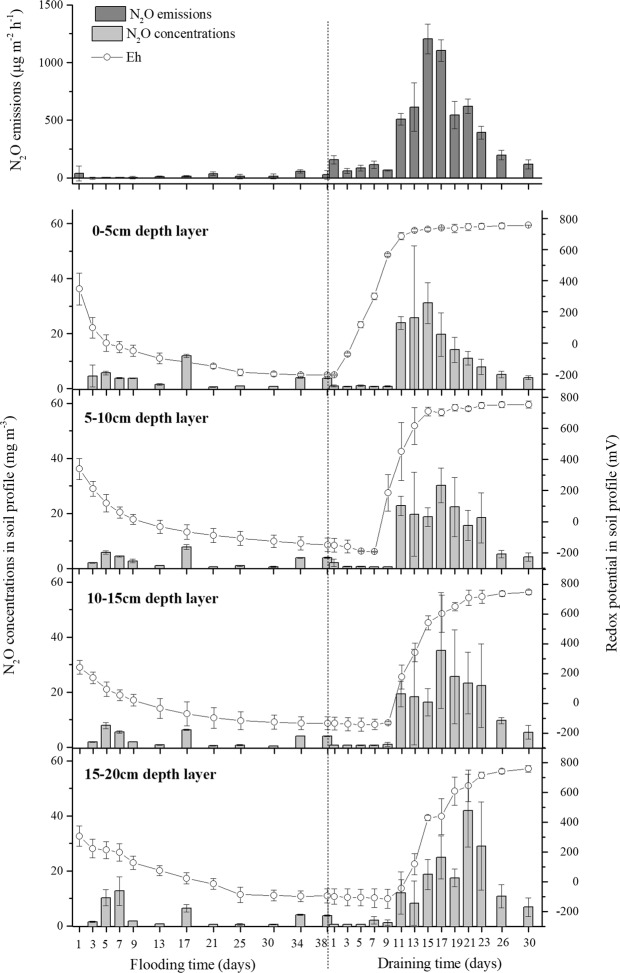
(Wei) Different patterns of N_2_O emission from soil surface (dark grey bars), N_2_O concentrations in soil profile (light grey bars) and soil redox potential in soil profile (dotted line) during flooding and draining periods. Data are mean ± standard error of three replicates.

The patterns of N_2_O concentrations in each depth layer and N_2_O emissions during flooding period were considerably different from that during drainage period (Fig. 1). The N_2_O emissions under flooding conditions maintained stable and low rates (below 56.89 µg m^−2^ h^−1^). In addition, N_2_O uptake was monitored several times over the course of the flooding period, and the highest N_2_O negative emission was −16.20 µg m^−2^ h^−1^ at flooding day 38. In contrast, significantly elevated N_2_O emissions were recorded during drainage period. The N_2_O emissions were lower at the beginning of draining process until the N_2_O emission continuously increased from draining day 10. The peak N_2_O emission, which occurred on draining day 15, was 1204.81 µg m^−2^ h^−1^ and then the levels continued to decrease during the following drainage period.

Similar to the N_2_O emission pattern, the N_2_O concentrations in each depth layer during drainage period were also much higher than they were during flooding. Under flooding conditions, the N_2_O concentrations in the whole soil profile fluctuated at around 7.00 mg N_2_O m^−3^ within a narrow range, except on flooding day 1. Furthermore, there was no significant difference in the N_2_O concentration among four depth soil layers during this period (Table 1). During the drainage period, all four depth layers showed negligible N_2_O concentrations (<2.0 mg N_2_O m^−3^) until draining day 11. Then, the N_2_O concentrations in four depth layers increased continually to their peak values at different draining days. The N_2_O concentrations of the 0–5 cm layer peaked on draining day 15, 5–10 cm and 10–15 cm layers peaked on draining day 17, and the 15–20 cm layer peak value was on draining day 21. Furthermore, the peak N_2_O concentrations in the 0–5, 5–10, 10–15, and 15–20 cm depth layers were 31.14, 30.22, 35.16, and 41.92 mg N_2_O m^−3^, respectively, and the patterns of the four depth layers were not significantly different during the draining period (Table 1).

**Table 1:** The variances of repeated measures of N_2_O dynamics and soil properties during flooding and drainage periods.

	Flooding period						Drainage period					
	Flooding Time		Soil depth Layer		Flooding Time × Soil depth Layer		Drainage Time		Soil depth Layer		Drainage Time × Soil depth Laye	
	F	*P*	F	*P*	F	*P*	F	*P*	F	*P*	F	P
N_2_O concentration	74.59	0.000	3.386	0.074	6.939	0.000	21.347	0.000	0.370	0.777	1.818	0.134
Eh	249.14	0.000	6.298	0.017	3.286	0.013	537.972	0.000	75.263	0.000	18.970	0.000
NH_4_^+^-N content	44.574	0.000	12.113	0.002	3.704	0.049	50.772	0.000	22.447	0.000	17.106	0.000
NO_3_^−^-N content	532.128	0.000	98.961	0.000	9.321	0.000	100.245	0.000	57.955	0.000	42.498	0.000
WFPS	19.829	0.000	38.203	0.000	4.260	0.009	25.021	0.000	6.736	0.014	2.796	0.047
Arch-*amoA* abundance	4.918	0.022	10.942	0.003	1.729	0.178	44.770	0.000	21.127	0.000	12.575	0.002
Bac-*amoA* abundance	8.549	0.003	17.762	0.001	6.264	0.002	38.325	0.000	27.257	0.000	14.889	0.000
narG abundance	2.483	0.115	31.238	0.000	3.817	0.015	0.869	0.438	37.163	0.000	8.829	0.000
nirK abundance	3.642	0.050	346.819	0.000	4.752	0.006	48.502	0.000	683.665	0.000	10.761	0.000
nirS abundance	22.682	0.000	20.685	0.000	2.402	0.076	2.723	0.096	21.799	0.000	0.933	0.498
nosZ abundance	0.316	0.608	75.849	0.000	2.017	0.182	0.568	0.479	16.111	0.001	0.409	0.758

*Unites of soil parameters are:* N_2_O *concentration* (mg N_2_O m^−3^), *Eh* (mV), NH_4_^+^-N content (mg kg^−1^ soil), NO_3_^−^-N content (mg kg^−1^ soil), and *WFPS* (%); *unites of various gene abundances are* copies g^−1^ dry soil.

### Properties of paddy soil columns during flooding and drying periods

An obvious variation was observed in the Eh of the entire soil column sample during the flooding and drainage periods (Fig. 1, Table 1). The soil Eh of the 0–20 cm layer rapidly fell during flooding process. The paddy soil draining caused a steep increased in the Eh with increasing draining time. However, the differences in Eh value varied significantly based on the drainage period in four depth layers (*P* < 0.001). The soil Eh of the 0–5 cm layer increased the fastest and was >540 mV at draining day 10, followed sequentially by the 5–10, 10–15, and 15–20 cm depth layers.

**Figure 2:**
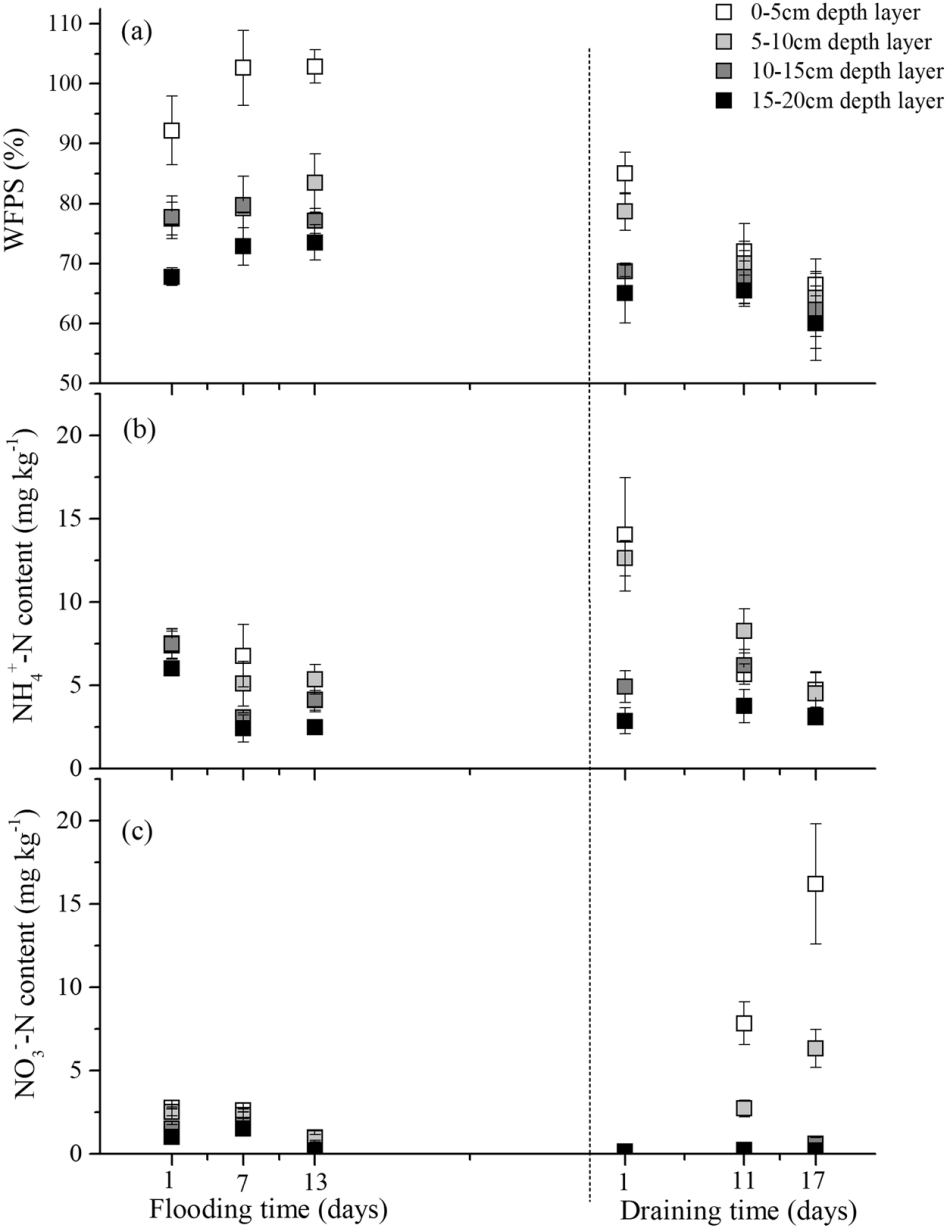
(Wei) Variations of (**a**) soil water, (**b**) ammonia-N (NH_4_^+^-N) content, and (**c**) nitrate-N (NO_3_^−^N) content in soil samples during flooding and draining incubation. Data are means ± standard error of three replicates.

The variation in the soil water content in 0–5 cm layer was more obvious than that in the other three layers during both flooding and draining periods (Fig. 2a). First, the soil water content of the 0–5 cm depth layer was 92% WFPS after 12 h flooding, and it kept increasing to 103% WFPS in the following flooding period. In contrast, the water content of the other three depth layers remained at much lower levels. During draining, the water content of the paddy soil column of 0–5 cm layer immediately fell to 85% WFPS on draining day 1, and continuously decreased 22% over the following 17 days. In addition, the water contents of the 5–20 cm layers also decreased with increasing draining time, to 65% WFPS.

The initial inorganic nitrogen contents of the paddy soil samples were at low levels with mean values of 7.12 mg NH_4_^+^-N kg^−1^ and 1.96 mg NO_3_^−^-N kg^−1^ on flooding day 1 (Fig. 2b,c). The NH_4_^+^-N content in the four depth layers was consumed by approximately 44% over 13 flooding days, whereas the NO_3_^−^-N contents simultaneously decreased by 74%. Following the draining period, the paddy soil columns showed dramatically elevated NH_4_^+^-N contents in the topsoil layer with initial values of 9.97 and 7.27 mg kg^−1^ in 0–5 cm and 5–10 cm layers, respectively during the 12 h draining process. However, the ammonium concentrations in the 0–10 cm layer reduced continuously with a prolonged draining period, and the bottom soil layer exhibited steady ammonium concentrations during the 17 draining days. On the contrary, the NO_3_^−^-N contents in the soil were very low on draining day 1 at 0.13 mg kg^−1^. Then, the nitrate concentrations in the 0–10 cm layer increased significantly during the subsequent draining period (*P* < 0.001), while that in the lower layer increased slightly.

### Quantitive changes of soil nitrifying and denitrifying microbes during flooding and drying

**Figure 3:**
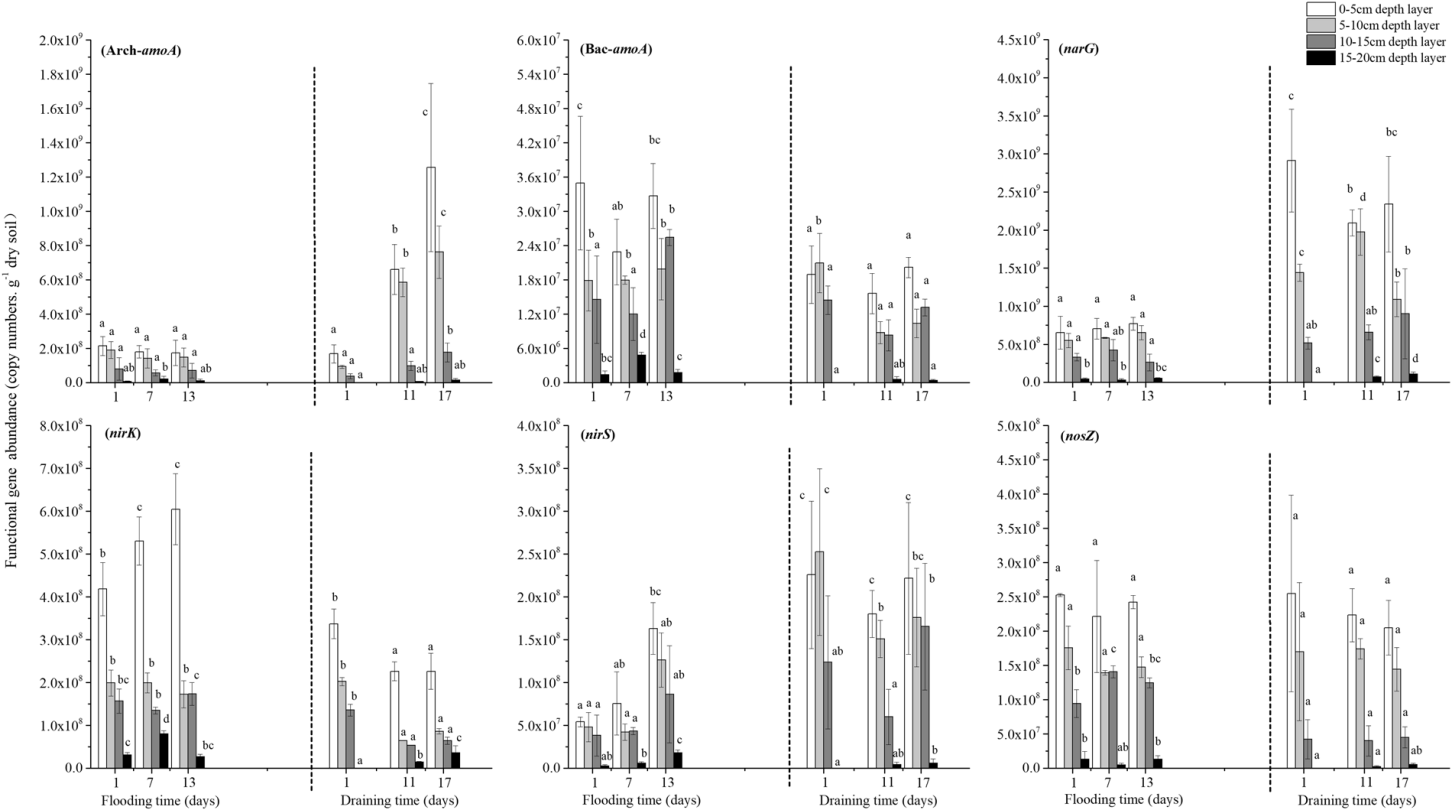
(Wei) Dynamics of functional gene abundance in soil during flooding and draining incubation. Data are means ± standard error of three replicates. Lowercase letters above bars indicate significant difference (*P* < 0.05) of time course according to ANOVA analyses.

It is clear from the results that the abundance of ammonia oxidizers (*Arch-amoA* and *Bac-amoA*) and denitrifiers (*narG*, *nirK*, *nirS* and *nosZ*) showed similar distributions with soil depth over the entire flooding-draining process (Table 1). Specifically, the highest gene copy numbers were observed in 0–5 cm depth layer, sequentially followed by the 5–10, 10–15, and 15–20 cm layers (*P* < 0.01). However, the responses of these functional genes to water conditions caused by flooding or draining differed greatly (Fig. 3).

AOA and AOB can both oxidize ammonium. In the paddy soil, the numbers of *Arch-amoA* gene copies were one to two orders of magnitude higher than those of the *Bac-amoA* gene at all incubation times. Under flooding conditions, there was no significant change in the *Arch-amoA* and *Bac-amoA* abundance of each depth layer. Following draining, the *Arch-amoA* gene copies in the paddy soil column samples remained unchanged initially. Then, the *Arch-amoA* gene numbers in the 0–5 cm and 5–10 cm layers increased sharply with prolonged draining (*P* < 0.001), whereas the levels in the lower soil layer increased slightly. In total, the 0–5, 5–10, 10–15, and 15–20 cm layers showed 6.45, 7.11, 3.70, and 1.30 times higher numbers of gene copies respectively, during the 17-day draining period. In contrast to the *Arch-amoA*, the *Bac-amoA* gene abundance in the soil declined immediately as the draining period commenced. Furthermore, the decline was maintained at a relatively steady state during the following draining period.

The *narG*, *nirK*, *nirS* and *nosZ* genes are the key indicators of denitrification. The present results showed no significant changes occurred in *narG* and *nosZ* gene abundance in the soil during the flooding process, while the *nirK* and *nirS* gene copies in 0–5 cm layer were increased significantly with flooding time. During draining process, the *narG* and *nirS* gene numbers exhibited a sharp increase in the 0–10 cm soil layer immediately after draining for 12 h. Then, the *narG* and *nirS* gene copies in soil column fluctuated slightly during the 17 days of the draining incubation. The *nirK* gene abundances during draining process were significantly lower than in flooding status, and decreased continuously with draining time. Furthermore, the *nosZ* gene abundance in the soil did not differ between the draining and flooding periods.

### Correlation analysis by Spearman test

Because of the significant N_2_O production and emission pattern under draining conditions, the correlations of soil variables and functional microorganisms with N_2_O in this process were analyzed only (Table 2). During the draining condition, the *Arch*-*amoA* gene involved in nitrification was the only population related to the N_2_O concentrations in the soil profile (*P* < 0.01), whereas the abundance of the other nitrifying and denitrifying populations (*Bac-amoA*, *narG*, *nirK*, *nirS*, and *nosZ*) were not correlated. The N_2_O concentrations in the soil profile were also positively linked to soil NO_3_^−^-N content and soil Eh (*P* < 0.01), while negatively correlated to soil WFPS (*P* < 0.05). As to the correlation of N_2_O emission from soil surface and soil functional microorganisms in each depth layer, the *Arch*-*amoA* gene in 0–20 cm layers was related to the N_2_O emission (*P* < 0.01), and also the *narG*, *nirK*, *nirS*, and *nosZ* gene copies in 15–20 cm layer were significantly correlated to N_2_O emission (*P* < 0.05). In addition, negative correlations were found between the N_2_O emission and the nitrification substrate of the soil NH_4_^+^-N content and soil WFPS in 0–10 cm depth layers (*P* < 0.01). And positive correlations were found between the N_2_O emission and the nitrification product of the soil NO_3_^−^-N in 0–15 cm soil layers, as well as the soil Eh in whole soil profile (*P* < 0.01). Moreover, the N_2_O emission had stronger correlations to soil properties (NO_3_^−^-N, NH_4_^+^-N, WFPS, and Eh) in the topsoil layers than it did in the lower depth layers during the draining period.

**Table 2:** Correlation coefficients between soil N_2_O concentration, N_2_O emission, and specific soil variables after draining paddy soil columns tested using Spearman index.

N_2_O	Soil depth layer	NO_3_^−^-N content	NH_4_^+^-N content	WFPS	Eh	*Arch-amoA* abundance	*Bac-amoA* abundance	*narG* abundance	*nirK* abundance	*nirS* abundance	*nosZ* abundance
N_2_O concentration^†^		**0.74** ^**^	−0.15	**−0.33** ^*^	**0.67** ^**^	**0.54** ^**^	−0.05	0.17	−0.01	0.14	0.21
N_2_O emission^‡^	0–5 cm	**0.85** ^**^	**−0.93** ^**^	**−0.95** ^**^	**0.82** ^**^	**0.88** ^**^	0.22	−0.47	−0.65	−0.10	−0.42
	5–10 cm	**0.92** ^**^	**−0.97** ^**^	**−0.80** ^**^	**0.68** ^*^	**0.83** ^**^	−0.62	−0.47	−0.45	−0.37	−0.13
	10–15 cm	**0.88** ^**^	−0.55	−0.48	**0.67** ^*^	**0.88** ^**^	−0.32	0.28	−0.43	−0.30	−0.05
	15–20 cm	0.57	0.15	−0.52	**0.78** ^*^	**0.68** ^**^	0.58	**0.83** ^**^	**0.88** ^**^	**0.70** ^*^	**0.83** ^**^

^*^Correlation is significant at the 0.05 level (2-tailed), **Correlation is significant at the 0.01 level (2-tailed.

^†^Correlations of N_2_O concentrations in four depth layers together with the other parameters in corresponding depth layers.

^‡^Correlations of N_2_O emission together with the other soil properties in each soil layer.

*Note*: *Unites of soil parameters are: N*_*2*_*O concentration* (mg N_2_O m^−3^), *N*_*2*_*O emission* (µg N_2_O m^−2^ h^−1^), *Eh* (mV), NH_4_^+^-N content (mg kg^−1^ soil), NO_3_^−^-N content (mg kg^−1^ soil), and *WFPS* (%); *unites of various gene abundances are* copies g^−1^ dry soil.

## Discussion

In the rice-growing season, paddy fields are often subjected to anthropogenic submergence and drainage several times to maximum the rice yields, while the traditional dry soil conditions of the fallow paddies would be acutely susceptible to heavy or prolonged rain and natural evaporation^6,20^. Different water conditions affect the biogeochemistry of paddy soils with respect to N_2_O production and emission^24^. Many studies have found that the most N_2_O was emitted from the paddy soils subjected to flooding-drying practice and the least N_2_O would degas from continuously flooded paddy soil either during cropping or fallow seasons^6,13,17,21^. In our study, we clearly detected very low levels of N_2_O production in each depth layer and N_2_O emission rates during the flooding period whereas the levels were significantly elevated in this fallow paddy soils after draining (Fig. 2). The average value of the N_2_O emission during draining period was 23.06 times higher than it was during the flooding period, and the peak N_2_O emission was 1204.81 µg m^−2^ h^−1^ after 15 days draining by evaporation.

When the submerged soil column was draining, O_2_ was able to infiltrate the soil matrix with water evaporation, and the redox state of the paddy soil gradually tended from reduction to oxidation reaction^16^. In our study, the soil WFPS ranged from 85% to 60% and the Eh value increased from −136.54 mV to 586.92 mV during the 17 days of the draining period (Figs 2, 3a). During this transition process, the water condition (60–70% WFPS) was conducive to N_2_O production^8,9^, and both nitrification and denitrification should contribute to the N_2_O production and induce large N_2_O emissions^25^. However, the soil NO_3_^−^-N content in this study was only 0.13 mg kg^−1^ at the beginning of the draining period (Fig. 3b), and this low availability of the NO_3_^−^ substrate severely impeded the denitrifiers activity as well as N_2_O emission^26^. The nitrification substrate in the upper soil layers was 13.35 mg NH_4_^+^-N kg^−1^ (Fig. 3c), which was relatively sufficient for nitrifiers activity and would be activated by continuous water evaporation and O_2_ infiltration. In addition, this point was proven by the observation that the NH_4_^+^-N content in the soil was gradually depleted after draining, which was accompanied by a significant increase in the nitrification product (NO_3_^−^-N). The correlation analysis also showed that the N_2_O net production and emissions were more relevant to functional genes of the nitrifiers than those of the denitrifiers (Table 3). Therefore, we could speculate that nitrification might be the more dominant process in N_2_O net production and emission in this draining paddy soil.

**Table 3:** Thermal profiles and primers used for quantitative polymerase chain reaction (qPCR) of different functional genes.

Target group	Thermal profile	Primers^†^	Primer sequence^‡^ (5′-3′)	Fragment length/bp	Reference
Bac-*amoA*	95 °C, 2 m; 95 °C, 5 s, 55 °C, 30 s, 72 °C, 10 s, 40 cycles; 95 °C, 15 s, 60 °C, 15 s, 95 °C, 15 s	1 F 2 R	GGGGTTTCTACTGGTGGT CCCCTCKGSAAAGCCTTCTTC	491	^44^
Arch-*amoA*	95 °C, 2 m; 95 °C, 5 s, 55 °C, 30 s, 72 °C, 10 s, 40 cycles; 95 °C, 15 s, 60 °C, 15 s, 95 °C, 15 s	23 F 616 R	ATGGTCTGGCTWAGACG GCCATCCATCTGTATGTCCA	624	^45^
*narG*	95 °C, 30 s; 95 °C, 5 s, 60 °C, 30 s, 72 °C, 10 s, 40 cycles; 95 °C, 15 s, 60 °C, 15 s, 95 °C, 15 s	571 F 773 R	CCGATYCCGGCVATGTCSAT GGNACGTTNGADCCCCA	203	^43^
*nirK*	95 °C, 30 s; 95 °C, 5 s, 60 °C, 30 s, 72 °C, 10 s, 40 cycles; 95 °C, 15 s, 60 °C, 15 s, 95 °C, 15 s	876 F 1055 R	ATYGGCGGVCAYGGCGA GCYTCGATCAGRTTRTGGTT	165	^46^
*nirS*	95 °C, 30 s; 95 °C, 5 s, 60 °C, 30 s, 72 °C, 10 s, 40 cycles; 95 °C, 15 s, 60 °C, 15 s, 95 °C, 15 s	cd3aF R3cd	GTSAACGTSAAGGARACSGG GASTTCGGRTGSGTCTTGA	425	^47^
*nosZ*	95 °C, 30 s; 95 °C, 5 s, 60 °C, 30 s, 72 °C, 10 s, 40 cycles; 95 °C, 15 s, 60 °C, 15 s, 95 °C, 15 s	1126 F 1381 R	GGGCTBGGGCCRTTGCA GAAGCGRTCCTTSGARAACTTG	256	^48^

^†^F and R, indicate forw ard and reverse primers, respectively.

^‡^Y = C or T; R = A or G; D = A, G or T; V = A, C or G; B = C, G or T; N = A, C, T or G; M = A or C.

Ammonium-oxidizing microorganisms (AOM) play vital roles in catalyzing the first step of nitrification, consists of AOB^27^ and AOA^28^. In our study, the *amoA* gene copies of AOA in the paddy soil columns were one to two orders of magnitude higher than those of the AOB during the entire draining process (*P* < 0.01), demonstrating that the AOA were more abundant nitrifiers in this paddy soil column. However, the numerical dominance also indicates a functional advantage to what extent and which kind of population is responsible for nitrification or N_2_O emissions are still open questions. Although both bacteria and archaea have ammonium oxidizing functions, their ecological niches are very different, and many crucial intermediates in their ammonium oxidation are not common^29^. These discrepancies result in the inequivalent contributions of AOA and AOB to N_2_O emissions. In our study, the *amoA* gene abundance of AOA and AOB showed different responses to the draining period. The N_2_O concentrations and N_2_O emissions were intensively correlated to *Arch-amoA* abundance in the entire soil column (both showed R = 0.88, P < 0.01), whereas no significant relationship was observed between N_2_O concentrations/emissions and *Bac*-*amoA* abundance (Table 3), indicating the more important role of archaea in the process of ammonia oxidation during this draining period. Previous studies have demonstrated that under extremely low concentrations of ammonium substrate, AOA played important roles in the N cycles^30^. For example, the abundance of AOA was much more than AOB at very low ammonium concentration in the open ocean (≤10 µM), and AOA abundance also exhibited more intense correlation to nitrification rates^31–33^. A preference of AOA for low NH_4_^+^ concentrations has also been reported from several environmental studies, especially for soil environments^30,34,35^. The NH_4_^+^ and NO_3_^−^ concentration in this paddy soil sample were very low at the beginning of the draining process at only 8.63 mg NH_4_^+^-N kg^−1^ soil and 0.13 NO_3_^−^-N kg^−1^ soil on average (Fig. 3b,c). Such a low availability of inorganic nitrogen might restrict the AOB activities, while AOA were highly adapted to this energy-stressed environments in contrast to bacteria^36^. Moreover, it has been previously hypothesized that the oxygen or carbon dioxide in the exudates of paddy soil affected AOA more than they did AOB^37^. Therefore, the significant increase in *Arch*-*amoA* abundance rather than *Bac*-*amoA* abundance in our study might be attributed to the higher affinity of AOA for oxygen increase in the process of draining soil.

**Figure 4:**
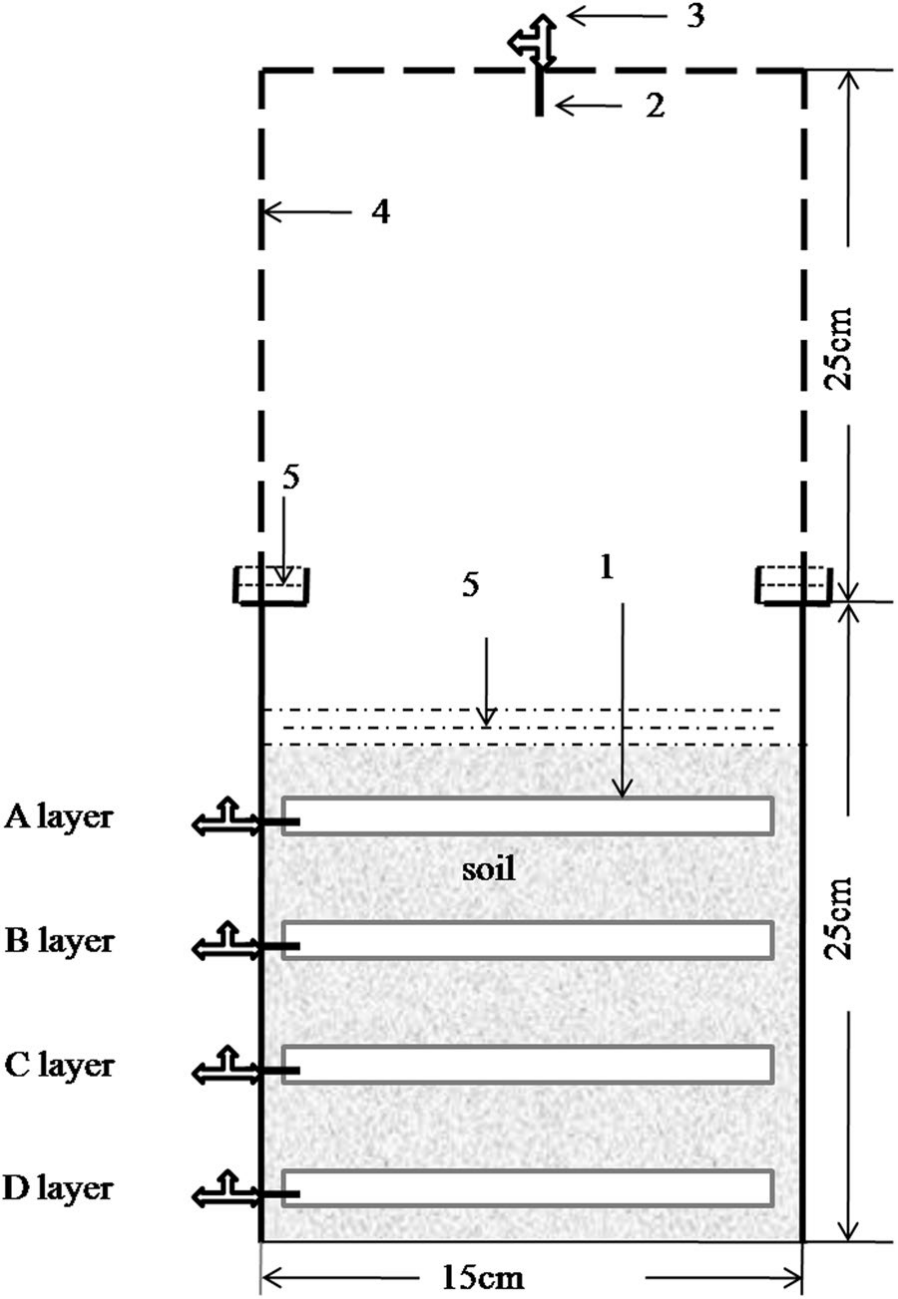
(Wei) Schematic diagram of the pot for gas sampling. (1) silicon tubes, (2) stainless steel tube, (3) three-way stopcock, (4) gas sampling static chamber, (5) water. A layer was 0–5 cm depth layer; B layer was 5–10 cm depth layer; C layer was 10–15 cm depth layer; and D layer was 15–20 cm depth layer.

In addition, significant differences in functional genes abundance (especially *Arch-amoA*), as well as NH_4_^+^ and NO_3_^−^ concentration were observed along the vertical soil profile (*P* < 0.01), with a gradual decrease from the 0–5 cm layer to the 5–10, 10–15, and 15–20 cm layers (Figs 3, 4). Compared to the subsoil, the surface soil usually contains more soil nutrients and harbors a higher density of microorganisms^14,38,39^. As a result, the N_2_O production in the surface soil should be higher than that in the subsoil. However, the N_2_O concentrations in the 0–10 cm depth layer was not significantly different from that in the 10–20 cm depth layer during the draining period (*P* > 0.05) (Fig. 2). This abnormal phenomenon might have its attributable reasons. The N_2_O concentration detected in each depth layer was a collective result of the N_2_O production, migration, and consumption of this layer^40^. With water evaporation from soil matrix, the water content in the 0–10 cm layer decreased rapidly because of soil draining, whereas no obvious change occurred in the 10–20 cm layer. The N_2_O concentrations in the 0–5 cm depth layer showed the highest increment during draining day 10 and peaked first, followed by levels in the 5–10, 10–15, and 15–20 cm layers in that order. Furthermore, aeration of the upper soil layers was higher and distance of gas diffusion to the soil surface was shorter than that of the lower layers, which would facilitate the gaseous diffusion^41^. Therefore, we could speculate that the N_2_O produced in the upper soil layers likely contributed more to the N_2_O emissions than that in the lower soil layers did, resulting in more N_2_O accumulation in lower soil layers.

## Materials and Methods

The sampling field was a double-cropping rice paddy over 100 years old, located in Huanghua town, Hunan Province, China (28°14′08″N; 113°13′05″E). This paddy soil is developed from Quaternary red clay and has been classified as loamy clay (Hydragric Anthrosols)^42^. The soil samples for basic property determination were collected using a five-point sampling method on February 20, 2014, which was still the winter fallow period. Soil properties at the point of the sampling were pH (H_2_O) 5.11; NH_4_^+^-N, 2.57 mg kg^−1^; NO_3_^−^-N, 2.73 mg kg^−1^; and organic matter content, 30.50 g kg^−1^.

### Intact soil cores collection and device installation

The intact soil cores (15 cm diameter, 0–20 cm depth) were collected from the sampling paddy field by self-made device, which have been used in a previous study^39^. Intact soil cores were collected using PVC cylinders (15 cm diameter, 25 cm high), and one hole (1.6 cm diameter each) through cylinder wall was made at positions of 2.5, 7.5, 12.5 and 17.5 cm from the bottom of cylinder, respectively. In the field, sampling points were selected randomly, then plant residues above the soil surface were manually removed. Soil cores were dug by fitting into the PVC cylinders and trimming off outer soil with a spade. After the soil cores (0–20 cm) were removed from the field, the bottom was covered with a plate (PVC plate, 18 cm diameter) and the cylinder was wrapped with film to prevent water loss. In total, 24 intact soil cores were prepared.

In laboratory, the bottom plates were sealed to each cylinder with glue. After that, a horizontal tunnel was made across the soil cores with a stainless tube (1.6 cm diameter) through each hole in the PVC cylinder. Then a silicon tube (14 cm long, 1.2 cm internal diameter, 0.2 cm wall thickness) closed with silicone septa at both ends, was inserted into each tunnel of the soil cores. One end of the stainless steel tube (3 cm long, 2 mm diameter) was inserted into the silicon tube through the septa and the other end was connected to a three-way stopcock outside the cylinder (Fig. 4). At last the holes in PVC wall were sealed with glue and the space between the soil columns and PVC cylinders was filled with soil slurry.

### Soil incubation

The entire incubation consisted of flooding period (1–38 days) and draining period (1–30 days). Three soil cores were selected randomly for gas sampling, three for redox potential (Eh) monitoring, and 18 for soil sampling at six time points. Thus, each treatment had three experimental replicates. Then, 500 mL double distilled water was added to each soil core from the soil surface, and 2 cm of the free water layer was maintained throughout the flooding period. After the flooding incubation, the free water above the soil surface was removed using a syringe, and the soil cores were drained by air evaporation for approximately 30 days. The pots were constantly incubated at 28 °C.

### Gas sampling and determination

Gas samples were collected on flooding day 1, 3, 5, 7, 9, 13, 17, 21, 25, 30, 34, 38, and draining day 1, 3, 5, 7, 9, 11, 13, 15, 17, 19, 21, 23, 26, 30. Using a static chamber method, two gas samples (30 mL) were first collected at 9:00 a.m. and 10:00 a.m. on each sampling day. And then the gas samples (5 mL) were collected from the silicon tubes in 0–5 cm, 5–10 cm, 10–15 cm, and 15–20 cm depth layer (Fig. 1), and an equal volume of helium was injected back into the tubes afterwards. The N_2_O concentrations of gas samples were determined using a gas chromatograph (Agilent 7890 A, Agilent Technologies, Santa Clara, CA, USA) fitted with an electron capture detector (ECD) for N_2_O analyses at 350 °C. In addition, the N_2_O emission/h (the N_2_O cumulative emission per hour) was calculated (expressed in µg N_2_O m^−2^ h^−1^) referred to the formula established by previous study^39^. The N_2_O concentrations (net prodcution) in each soil layer were expressed as milligram N_2_O per cubic meter (mg N_2_O m^−3^).

### Soil sampling and measurements

Soil samples were collected on flooding day 1, 7, 13, and draining day 1, 11, 17, representing the initial, increasing, and peak phases of N_2_O emission during the draining period (based on the pre-experiment) and their corresponding time in the flooding period. The soil sampler consisted of two, longitudinally split stainless steel tubes (25 cm long, 4 cm inside diameter), connected to each other by rubber belts. Prior to sampling the flooding soils, the surface water layer was first removed, and then the sampler was inserted vertically into the soil column to obtain a 20-cm-long soil sample. Then, each soil sample was immediately divided into four parts as follows: 0–5, 5–10, 10–15, and 15–20 cm depth, respectively. This procedure was repeated three times with each soil column, and then two portions of the mixed soil samples were divided, and one was stored at −80 °C for further molecular analysis while the other portion was stored at 4 °C for chemical property analysis.

The soil water content was analyzed using the oven-drying method, and all results were converted to water-filled pore space (WFPS) units. The soil substrate availability of nitrate-N (NO_3_^−^-N) and ammonia-N (NH_4_^+^-N) was determined by extracting the samples with 2 M potassium chloride (KCl) solution, followed by analysis using a continuous-flow injection analyzer (FIAstar 5000, Foss Corporation, Hillerod, Denmark). The soil Eh value was monitored using redox electrodes (FJA-3, Nanjing, China), which were inserted vertically at depths of 2.5, 7.5, 12.5 and 17.5 cm before flooding treatment.

### DNA extraction

Soil microbial DNA was extracted from 0.3 g soil and stored at −80 °C using a previously described method^43^. The DNA quality and concentration were measured using a NanoDrop NA-1000 spectrophotometer (Nanodrop Technologies, Wilmington, DE, USA). Each soil sample was extracted three times, pooled, and then stored at −80 °C for further analysis.

### Quantitative polymerase chain reaction (qPCR)

The forward (F) and reverse (R) primer pairs amoA-23F/amoA-616R, amoA-1F/amoA-2R, narG-571F/narG-773R, nirK-876F/nirK-1055R, nirS-cd3aF/nirS-R3cd, and nosZ-1126F/nosZ-1381R were used to quantify the ammonia monooxygenase gene of archaea (*Arch-amoA*) and bacteria (*Bac-amoA*), nitrate reductase gene (*narG*), nitrite reductase gene (*nirK* and *nirS*), and nitrous oxide reductase gene (*nosZ*). The primer sequences and thermal conditions used for the analysis are listed in Table 3. The quantitative polymerase chain reaction (qPCR) assay was performed using an ABI 7900HT (Applied Biosystems, Foster City, CA, USA). The 10 μL reaction mixtures consisted of 5 μL 2× SYBR green mix II (Takara Biotechnology Co. Ltd., Dalian, China), 0.2 μL 50× Rox Reference Dye (Takara Biotechnology Co. Ltd., Dalian, China), 0.3 μL (0.4 μmol L^−1^) each of the F and R primers, and 5 ng sample DNA template. Standard curves were constructed using a series of 10-fold dilutions of linearized plasmids containing the target gene. Three parallel PCR replicates of all samples were performed on each plate. A melting curve analysis was conducted following the assay to verify the specificity of the amplification product. The PCR efficiency was in the range of 90–110%.

### Data treatment and statistical analysis

Given that the depth within cores and the core values over time are not independent, the repeated measures analysis of variance was employed to compare the effects of soil depth and incubation time (flooding time and draining time respectively) on N_2_O, physical and chemical properties, and functional gene abundances (Table 2). The variance of functional gene abundances in each depth layer over incubation time were analyzed using one-way ANOVA method, and least significant difference (LSD) test was used to evaluate the effects of the above ANOVA tests. Due to the nonnormal distribution of the soil NO_3_^−^ concentrations after draining (including the transformed data), spearman’s correlation was used to analyze the relationship of soil variables and N_2_O concentrations and emissions after draining. A *P* < 0.05 was considered significant, and all the statistical analyses were performed using the statistical package for the social sciences (SPSS) 13.0 (SPSS Inc., USA).

## Supplementary information

Dataset 1

Dataset 2

## Data Availability

The datasets generated and/or analysed during the current study are available in the [Dataset1.xlsx, Dataset2.xlsx] repository.
